# Tissue Culture-Induced Heritable Genomic Variation in Rice, and Their Phenotypic Implications

**DOI:** 10.1371/journal.pone.0096879

**Published:** 2014-05-07

**Authors:** Di Zhang, Zhenhui Wang, Ningning Wang, Yang Gao, Ying Liu, Ying Wu, Yan Bai, Zhibin Zhang, Xiuyun Lin, Yuzhu Dong, Xiufang Ou, Chunming Xu, Bao Liu

**Affiliations:** 1 Key Laboratory of Molecular Epigenetics of the Ministry of Education (MOE), Northeast Normal University, Changchun, China; 2 Faculty of Agronomy, Jilin Agricultural University, Changchun, China; 3 Jilin Academy of Agricultural Sciences, Changchun, China; Ben-Gurion University, Israel

## Abstract

**Background:**

Somaclonal variation generally occurs in plants regenerated from tissue culture. However, fundamental issues regarding molecular characteristics, mutation rates and mutation spectra of plant somatic variation as well as their phenotypic relevance have been addressed only recently. Moreover, these studies have reported highly discrepant results in different plant species and even in the same plant genotype.

**Methodology/principal findings:**

We investigated heritable genomic variation induced by tissue culture in rice by whole genome re-sequencing of an extensively selfed somaclonal line (TC-reg-2008) and its wild type (WT) donor (cv. Hitomebore). We computed the overall mutation rate, single nucleotide polymorphisms (SNPs), small scale insertions/deletions (Indels) and mobilization of transposable elements (TEs). We assessed chromosomal distribution of the various types of genomic variations, tested correlations between SNPs and Indels, and examined concomitancy between TE activity and its cytosine methylation states. We also performed gene ontology (GO) analysis of genes containing nonsynonymous mutations and large-effect mutations, and assayed effects of the genomic variations on phenotypes under both normal growing condition and several abiotic stresses. We found that heritable somaclonal genomic variation occurred extensively in rice. The genomic variations distributed non-randomly across each of the 12 rice chromosomes, and affected a large number of functional genes. The phenotypic penetrance of the genomic variations was condition-dependent.

**Conclusions/significance:**

Tissue culture is a potent means to generate heritable genetic variations in rice, which bear distinct difference at least in space (chromosomal distribution) from those occurred under natural settings. Our findings have provided new information regarding the mutation rate and spectrum as well as chromosomal distribution pattern of somaclonal variation in rice. Our data also suggest that rice possesses a strong capacity to canalize genetic variations under normal growing conditions to maintain phenotypic robustness, which however can be released by certain abiotic stresses to generate variable phenotypes.

## Introduction

It has been well-established that plants possess the ability to regenerate from totipotent, differentiated somatic cells via *in vitro* tissue culture. However, this process can be associated with a variety of genetic and epigenetic instabilities, and some of which can be translated into heritable phenotypic variations, a phenomenon has been collectively termed somaclonal variation [1]. Since the first report of somaclonal variation in regenerated plants from sugarcane tissue culture [2], numerous cases of somaclonal variation involving a large number of plant species have been reported [3]. However, insights into the molecular nature, mutation rate and spectrum of somaclonal variation at the molecular level (nucleotide sequence changes) have been gained only recently thanks to the revolutionary breakthroughs of new analytical tools, especially the next generation sequencing (NGS)-based genomic analysis.

Hitherto, three whole genome sequencing-based studies on plant somaclonal variation have been published, one in *Arabidopsis* [4] and two in rice [5], [6]. The *Arabidopsis* study revealed that regenerated plants (regenerants) displayed a generally elevated genome-wide DNA sequence mutation rate from naturally occurred mutations, and which bore distinct molecular mutation spectra, with base substitutions being the primary type of genomic changes [4]. Unexpectedly, no transpositional reactivation of transposable elements (TEs) was identified in the regenerants of *Arabidopsis* [4], suggesting that TE transpositional activity, a widely believed causal factor for tissue culture-induced genetic variations [3], [5]–[7], is not underpinning somaclonal variations in *Arabidopsis*. In contrast, both nucleotide sequence changes and TE transpositions were detected in rice regenerants [5], [6]. In aspect of TE transpositional activity, however, the two rice studies have obtained sharply discrepant results [5], [6]. While at least 13 TE families were transposed causing 34 new insertions were reported by Sabot *et al.* [5], only a single TE (*Tos17*) was shown to be active, and which caused only 10 new insertions in the study by Miyao *et al.* [6]. Causes for the discrepancy remained elusive, but genetic context effect can be ruled out as both studies used the same rice genotype, Nipponbare. Clearly, additional studies with more robust analysis and independent validation are needed to reveal possible rules of the molecular nature and mutation spectra in general and TE mobility in particular for a deeper understanding of plant somatic variation, a phenomenon bears broad implications in germplasm *in vitro* conservation and propagation, germplasm innovation and biotechnology-facilitated crop improvement [3].

In this study, we conducted whole genome re-sequencing of a stabilized rice line, TC-reg-2008, which was originated from tissue culture of a *japonica* rice cultivar Hitomebore, and had been selfed for eight successive generations. We compared the whole genome of TC-reg-2008 with its original wild-type (WT) donor at single-base resolution from a genome-wide scale. We showed that extensive and heritable genomic variations including single nucleotide polymorphisms (SNPs), small-scale insertions and deletions (Indels), as well as transpositional reactivation of three TEs occurred in TC-reg-2008 in spite of the fact that this line showed no appreciable phenotypic variations compared to its WT donor under normal growing conditions. Our data have provided additional insights into the molecular features of somaclonal variation in rice and indicated their phenotypic canalization under normal conditions.

## Materials and Methods

### Plant Materials and Genomic DNA Extraction

The TC-reg-2008 and wild-type rice (*Oryza sativa* ssp. *japonica* cv. Hitomebore) were used for this study. Callus of Hitomebore were induced from germinating seeds on Murashige-Skoog solid medium containing 2 mg/L 2,4-dichlorophenoxyacetic acid (2,4-D) at 26±1°C in darkness for 1 month, then transferred into N6 liquid medium containing 1 mg/L 2,4-D and cultured at 26±1°C in darkness for five months. Then, embryogenic calli were subcultured on N6 medium with 0.3% casamino acid, 0.1% proline and 1 mg/L 2,4-D at 26±1°C under a 14 hrs photoperiod for plant regeneration, followed by rooting in the regeneration medium containing 0.1 mg/L 6 benzyladenine (6-BA) and 0.01 mg/L naphtaleneacetic acid (NAA). Finally, regenerated plantlets in healthy conditions were transferred to soil and grown in green house. Genomic DNA from leaf tissues of uncultured wild-type (WT) and TC-reg-2008 (selfed for eight consecutive generations) was isolated by a modified CTAB method and purified by phenol extractions. Genomic DNA was used for whole genome re-sequencing, Southern blotting, locus-specific PCR amplifications and bisulfite sequencing.

### Whole Genome Sequencing, Building of WT Reference and Identification of SNPs and Indels

Purified high quality DNA of both samples was prepared for whole genome re-sequencing. Library construction, cluster generation and Hiseq2000 sequencing were carried out with standard protocols. The 90 nt paired-end reads were yielded. Raw data were cleaned by removing adaptor contamination and low quality reads before further analysis. To identify genomic variations in TC-reg-2008, a whole genome WT reference sequence was generated by mapping-based method [8]. This was accomplished by mapping the WT reads against the standard Nipponbare reference genome (MSU 7.0 http://rice.plantbiology.msu.edu/index.shtml) [9] using Burrows Wheeler Aligner (BWA) [10]. The reads which were uniquely mapped and the mapping quality scores were higher than 30 (Phred scale) were used to produce a new theoretical reference genome named WT Reference for rice cv. Hitomebore, in which the sequence variations between WT and Nipponbare were replaced with WT of Hitomebore by a stringent custom Perl scripts. Thereafter, the reads of TC-reg-2008 were aligned to the Hitomebore WT Reference to identify SNPs and Indels using SAMtools (v0.1.5c) [11] with the following parameters: Phred score ≥30, coverage ≥10 and ≤100.

To explore the genome-wide patterns of genomic variation in the TC-reg-2008 genome relative to that of WT, a 100 kilobase pair (kb) sliding window along each of the 12 rice chromosomes was applied to calculate DNA polymorphisms using Perl scripts. Moreover, based on the Generic Feature Format Version 3 (GFF3) files of the annotated Nipponbare reference genome, all detected SNPs were annotated and calculated as genic or intergenic. SNPs in genic regions were classified as coding sequences (CDS), untranslated regions (UTR), and introns. According to the amino acid substitutions, SNPs in CDS were divided into two types, synonymous and nonsynonymous. The nonsynonymous and large-effect mutations were further analyzed for gene ontology (GO) enrichment using the AgriGO Singular Enrichment Analysis tool [12].

### Detection and Validation of New Insertions of Transposable Elements (TEs)

To investigate whole genome TE mobilization events, we used a modified paired-end method reported previously [5]. All known TE sequences were compiled to establish a TE library. A new TE reference was first generated by removing the known TEs from Nipponbare sequence (MSU 7.0 http://rice.plantbiology.msu.edu/index.shtml) [9], and then each end of TC-reg-2008 and its WT (Hitomebore) raw data were aligned against to this new TE reference and the TE library using BWA [10]. After that, the reads characterized by one end being highly matching to the TEs library and the other one mapping uniquely to this new TE reference were selected using SAMtools followed by perl scripts as candidate TEs. Furthermore, these candidate TEs were filtered and those uniquely appeared in TC-reg-2008 were retained, which were newly inserted TE events in TC-reg-2008.

The TE mobilization was verified by Southern blotting following rational reported previously [13]. Specifically, genomic DNA (∼10 µg per sample) was digested by *Xba*l (New England Biolabs Inc.), which was suitable for assessing copy number of the analyzed TEs as there were no restriction sites or only one site on one side of the probe region. Restricted DNA was run through 1% agarose gels and transferred onto Hybond N+ nylon membranes purchased from Amersham Pharmacia Biotech by the alkaline transfer method recommended by the supplier. Three pairs of primers were designed (see Table S1) to amplify the fragments from the WT genomic DNA used as hybridization probes. Authenticity of the probe fragments was verified by Sanger-sequencing, and labeled with fluorescein-11-dUTP by the Gene Images random prime-labeling module (Amersham Pharmacia Biotech). Hybridization signal was detected by the Gene Images CDP-Star detection module (Amersham Pharmacia Biotech) after washing at a stringency of 0.2× SSC, 0.1% SDS for 2×50 min. The blots were exposed to X-ray films for chemiluminecence signal detection. The insertion events by *Tos17* (the most active TE) in TC-reg-2008, were also investigated by locus-specific PCR amplifications and cloning/sequencing. Specifically, the five insertions by retrotransposon *Tos17* were analyzed by locus-specific PCR amplifications with primers targeting both the TE terminals and their immediate flanks (Table S2). The PCR reactions were performed under conditions of 10 min at 94°C; 35 cycles of 30 sec at 94°C, 45 sec at 58°C, 1.5 min at 72°C; a final extension for 10 min at 72°C. PCR products were visualized by ethidium bromide staining after electrophoresis through 2% agarose gels.

### Bisulfite Sequencing of Original *Tos17* Loci

The protocol was basically as reported [14], with minor modifications. Specifically, genomic DNA (∼0.5 µg) was treated by EZ DNA Methylation- Gold KIT (Zymo Research, http://zymosearch.com), and then amplified with designed PCR primers followed by nested PCR for *Tos17* on chromosome 10 and general PCR for *Tos17* on chromosome 7 (Table S3). The sequences were analyzed by the Kismeth program (http://katahdin.mssm.edu/kismeth/revpage.pl) and the Kismeth generated data were presented by histograms.

### Abiotic Stress Treatments

Uniform and healthy seeds of TC-reg-2008 and WT were selected for abiotic stress treatments, including 150 mM NaCl (salinity), 0.25 mM CuSO_4_ (heavy metal), 0.25 mM HgCl_2_ (heavy metal) and 1 mM sodium nitroprusside (nitric oxide). The seeds were disinfected and thoroughly rinsed, and then germinated in petri-dishes with sterile water at 28°C in the darkness for 2 days. The seeds (30 each for mock and treatment) were then transferred to an illumination incubator (30°C for18 hrs during the day and 25°C for 6 hrs during the night), and the stressed groups were watered with Hoagland solution containing four kinds of abiotic stress while the control group was watered with only Hoagland once each day for a week. Notably, the seeds for sodium nitroprusside stress were placed on an individual illumination incubator because sodium nitroprusside could release NO. All statistical analyses were done by R (v3.0.2) (http://www.r-project.org/).

## Results

### Tissue Culture Induced DNA Polymorphisms and Base Substitution Patterns

A tissue cultured-originated, and extensively selfed (for 8 successive generations) rice line named TC-reg-2008 was re-sequenced by the Illumina Higseq 2000 platform along with its WT donor (cv. Hitomebore). A salient feature of this line was that it did not show any discernible variation in phenotypes compared with its WT under normal controlled or paddy-field growing conditions, but showed clear phenotypic variation under several abiotic stresses. Therefore, all genomic changes occurred to this line should reflect heritable changes whose biological effects were efficiently canalized under normal conditions but which were released (penetrated) under certain stress conditions.

After removing contaminations and low quality reads, 12.4 G clean data were yielded in total, which were comprised of 318 million 90 nt paired-end reads. The genomic re-sequencing data were mapped against the standard rice genotype Nipponbare reference genome (MSU7.0 http://rice.plantbiology.msu.edu/index.shtml) [9]. Approximately 94% re-sequencing reads pairs of TC-reg-2008 and its WT (cv. Hitomebore) were successfully mapped to the Nipponbare reference genome, and of which more than 75% reads were uniquely mapped (Table S4). On average, the effective sequencing depths of unique mapped reads were 15.4× and 14.7× coverage for TC-reg-2008 and WT, respectively.

We compared the DNA polymorphisms including single nucleotide polymorphisms (SNPs) and small scale (≤2 bp) insertions or deletions (Indels) between the WT genome and that of TC-reg-2008 using SAMtools (v0.1.5c) [11]. A total of 54,268 DNA polymorphisms were detected, which included 37,332 SNPs and 16,936 small-scale Indels, both of which were mapped throughout the rice genome. The average mutation rate was 5.0×10^−5^ base substitutions per site across all the chromosomes. Based on the distribution of DNA polymorphisms, we found that both SNPs and Indels were non-randomly occurred across each of the 12 rice chromosomes (Table 1). Interestingly, the number of SNPs was significantly correlated with the number of Indels both at the whole genome level and for each of the 12 rice chromosomes (Figure S1). This suggests that probably a common mutational mechanism underlies the two kinds of genomic changes, or one kind mutation has increased propensity for the other to occur. The average density of DNA polymorphisms (defined as the average number of DNA polymorphisms observed for each 100 kb interval along each of all the 12 rice chromosomes) was 14.54, including 10 SNPs, 1.93 insertions and 2.6 deletions in TC-reg-2008 relative to WT (Table 1). The highest and lowest numbers of DNA polymorphisms per 100 kb were located to chromosome 11 (24.67) and 5 (3.5), respectively (Table 1). Within each chromosome, the peaks of SNPs and Indels exhibited the same trend (Figure S2), consistent with the overall strong correlation between the two kinds of changes (Figure S1). To test whether and to what extent the distribution of tissue culture-induced genomic variations would overlap with the genomic regions known as hyper-mutable in the course of rice evolution and domestication [15], we did a comparison and found that only two concentrated areas of DNA polymorphisms were located to these hyper-mutable genomic regions (Figure S2), suggesting possible different mutagenic mechanisms governing tissue culture-induced mutagenesis from that underlying natural genetic variations.

**Table 1:** DNA polymorphisms on individual chromosomes observed between TC-reg-2008 and its WT genomes.

Chr.	No. polymorphism	Polymorphism/100 kb	No. SNPs	SNP/100 kb	No. insert.	Insert./100 kb	No. delet.	Delet./100 kb
1	7874	18.20	5399	12.48	1057	2.44	1418	3.28
2	4753	13.23	3211	8.94	638	1.78	904	2.52
3	3111	8.54	1804	4.95	550	1.51	757	2.08
4	7154	20.15	4879	13.74	1046	2.95	1229	3.46
5	1050	3.50	400	1.34	208	0.69	442	1.48
6	3752	12.01	2626	8.40	455	1.46	671	2.15
7	4723	15.90	3279	11.04	636	2.14	808	2.72
8	5081	17.86	3811	13.40	521	1.83	749	2.63
9	1787	7.77	1163	5.05	223	0.97	401	1.74
10	3602	15.52	2541	10.95	473	2.04	588	2.53
11	7160	24.67	5329	18.36	824	2.84	1007	3.47
12	4221	15.33	2890	10.50	584	2.12	747	2.71
Total★/Average♦	54268★	14.54♦	37332★	10.00♦	7215★	1.93♦	9721★	2.60♦

★ Total number of polymorphisms: SNPs, insertions and deletions as a whole.

♦ Average polymorphisms: total SNPs, insertions and deletions per 100 kb.

**Figure 1:**
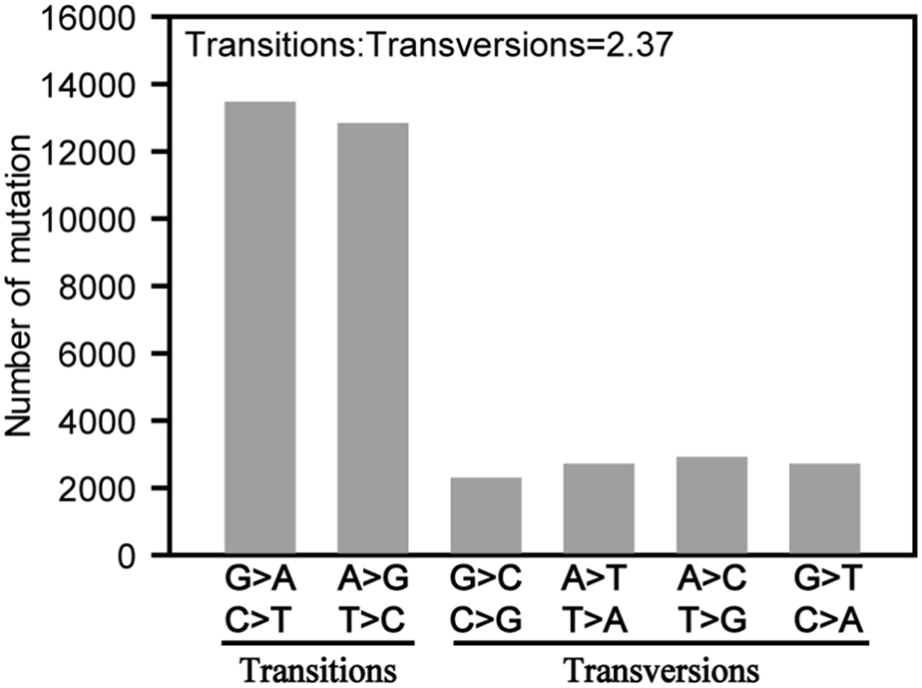
Classification of base substitution mutation between TC-reg-2008 and WT.

The identified SNPs were categorized and analyzed for their base substitution patterns. The average ratio of transitions (C/T or A/G) to transversions (C/G, T/A, A/C or G/T) in TC-reg-2008 vs. WT was 2.37 (Figure 1), which ranged from 1.70 for chromosome 4 to 2.76 for chromosome 6 (Table S5). The analysis of transitions and transversions suggested that C/T transition was the most frequently occurred base substitutions (Figure 1), which was consistent with previous results in both *Arabidopsis* [4] and rice [6]. Changes from A to G and A to C were also observed with high frequencies. However, the average ratio of transitions vs. transversions we detected (2.37) was substantially greater than those reported previously in both *Arabidopsis* [4] and rice [6], which were 0.92 and 1.1, respectively.

### Molecular Characteristics of the Tissue Culture Induced SNPs, Gene Ontology (GO) of the Affected Genes, and Evidence for Canalization

**Figure 2:**
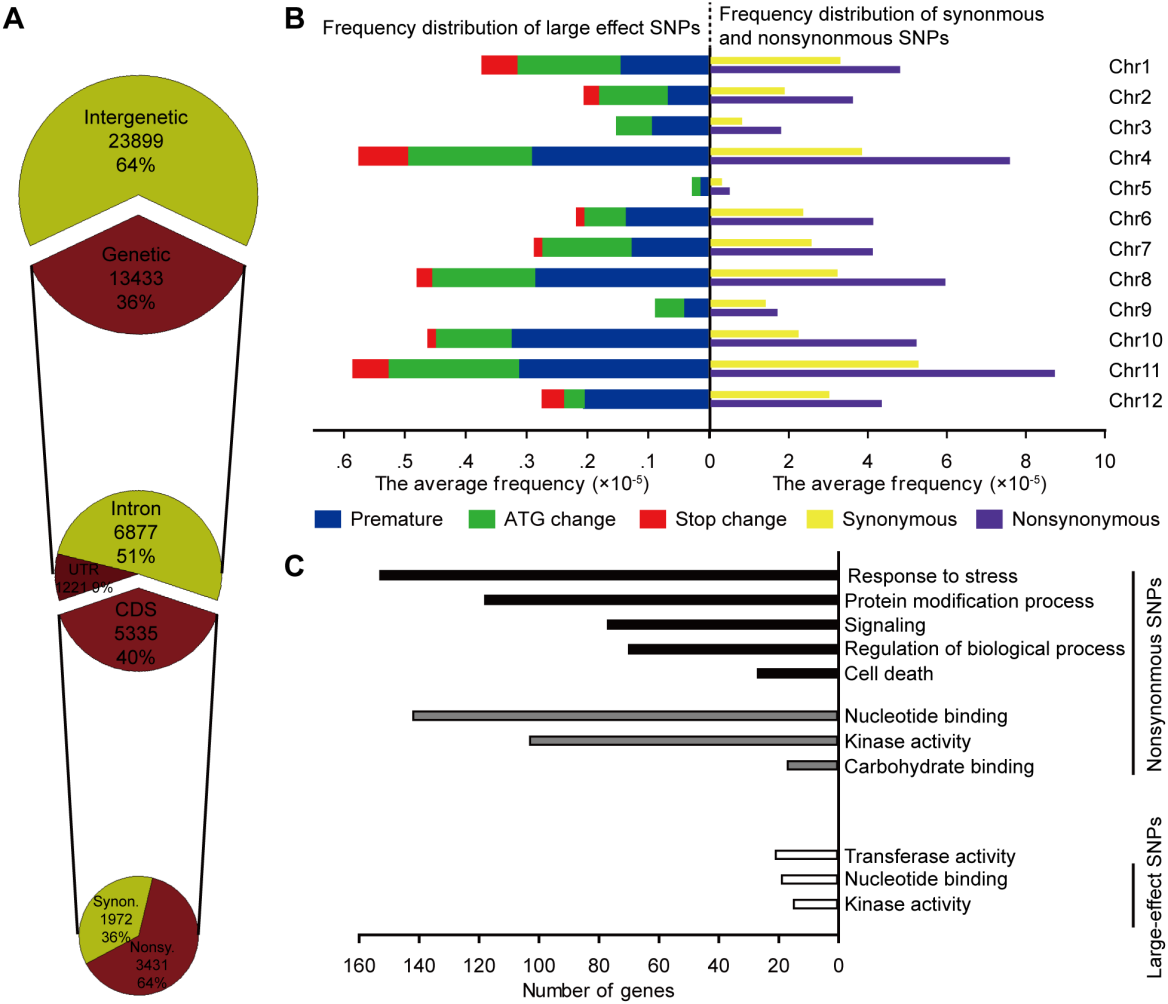
Annotation of homozygous SNPs between TC-reg-2008 and WT. (A) Number and percentile of homozygous SNPs between TC-reg-2008 and WT were located in genic and intergenic regions, and then SNPs located in the genetic regions were further annotated. (B) The average frequency distributions of synonymous, nonsynonymous and large effect SNPs on each of the 12 rice chromosomes. The *x*-axis represents the average frequency (×10^−5^) of synonymous, nonsynonymous and large effect SNPs on each chromosome. (C) Function assignment of nonsynonymous and large-effect SNPs related to gene categories. The black horizontal bars denote the number of nonsynonymous SNPs occurred to genes enriched in biological process. The grey horizontal bars indicate the number of nonsynonymous SNPs occurred to genes enriched in molecular function. The white horizontal bars indicate the number of large effect SNPs occurred to genes enriched in molecular function.

The majority of detected SNPs between TC-reg-2008 and WT were located in inter-genic regions (64%), while only 13,433 (36%) occurred in genic regions, consistent with possible selection constraint for the later. Of genic SNPs, 1,221 (9%), 6,877 (51%) and 5,335 (∼40%) were located in untranslated regions (UTR), introns, and gene-coding regions (CDS), respectively (Figure 2A). The SNPs located in CDS were further classified into nonsynonymous SNPs (totaling 3,431) and synonymous SNPs (totaling 1,972) (Figure 2A). Furthermore, 1,888 genes were found to contain more than one nonsynonymous SNP (Table S6). The average frequency of synonymous and nonsynonymous SNPs was significantly different among the 12 rice chromosomes (Figure 2B, right). There were distinctively more CDS SNPs of both synonymous and nonsynonymous types in chromosome 1, 4, 8 and 11 than the rest eight chromosomes (Figure 1B, right). We further analyzed large-effect SNPs in TC-reg-2008, which by definition, were those that contained substitutions that introduced premature termination codons (Premature, 132), eliminated translation initiation sites (ATG change, 120), and replaced nonsense by sense codons (Stop change, 27) (Table S7). We found that the average frequency of these large-effect SNPs was also non-randomly distributed among the 12 rice chromosomes, with>50% being mapped to the same four chromosomes, 1, 4, 8 and 11 (Figure 1B, left).

Based on Gene Ontology annotations, the 1,888 genes containing nonsynonymous SNPs could be classified into different categories. All mutations were enriched in two broad categories: biological process and molecular function. Regardless of broad categories, the three most enriched specific gene categories were “response to stress” (153), “nucleotide binding” (142) and “protein modification process” (118) (Figure 2C). Moreover, GO analysis for the large-effect mutations showed that they were enriched in the broad category of molecular function, which included enzymes and proteins known to participate in transferase activity (21), nucleotide binding (19) and kinase activity (15) (Figure 2C), underscoring functional consequences of these mutations.

The foregoing results indicated that extensive and trangenerationally inherited genomic variations occurred in TC-reg-2008 as a result of tissue culture (Table 1). Moreover, a large number of the mutations affected genes with important functions (Figure 2C). Unexpectedly, in spite of these biologically meaningful genetic mutations, TC-reg-2008 did not show appreciable phenotypic differences from its WT donor (cv. Hitomebore) under normal controlled condition or paddy-field trails (data not shown). Although a robust explanation for this conundrum is gene redundancy given that many genes in the rice genome, as in those of other higher eukaryotes, exist as gene families, we suspected that this could be also due to the well-known phenomenon of canalization or developmental robustness [16]–[20]. If this was the reason or one of the reasons, then phenotypic variability might be released in TC-reg-2008 under some stress conditions whereby canalization was perturbed [21]. To test this possibility, we subjected TC-reg-2008 together with WT to three kinds of abiotic stresses including salt, heavy metals (CuSO_4_ and HgCl_2_) and overdose nitric oxide (Figure S3A), and measured three phenotypic traits, shoot-length, root-length and total seedling fresh weight. We found that for each trait, TC-reg-2008 showed significant difference (inferior performance) from WT at least in one of the stress conditions (Figure S3B, C and D). These results suggest that the cryptic effects on phenotypes by the somaclonal genomic variations are due at least in part to canalization.

### Tissue Culture Induced TE Mobility and its Concomitancy with Cytosine Methylation Dynamics

**Figure 3:**
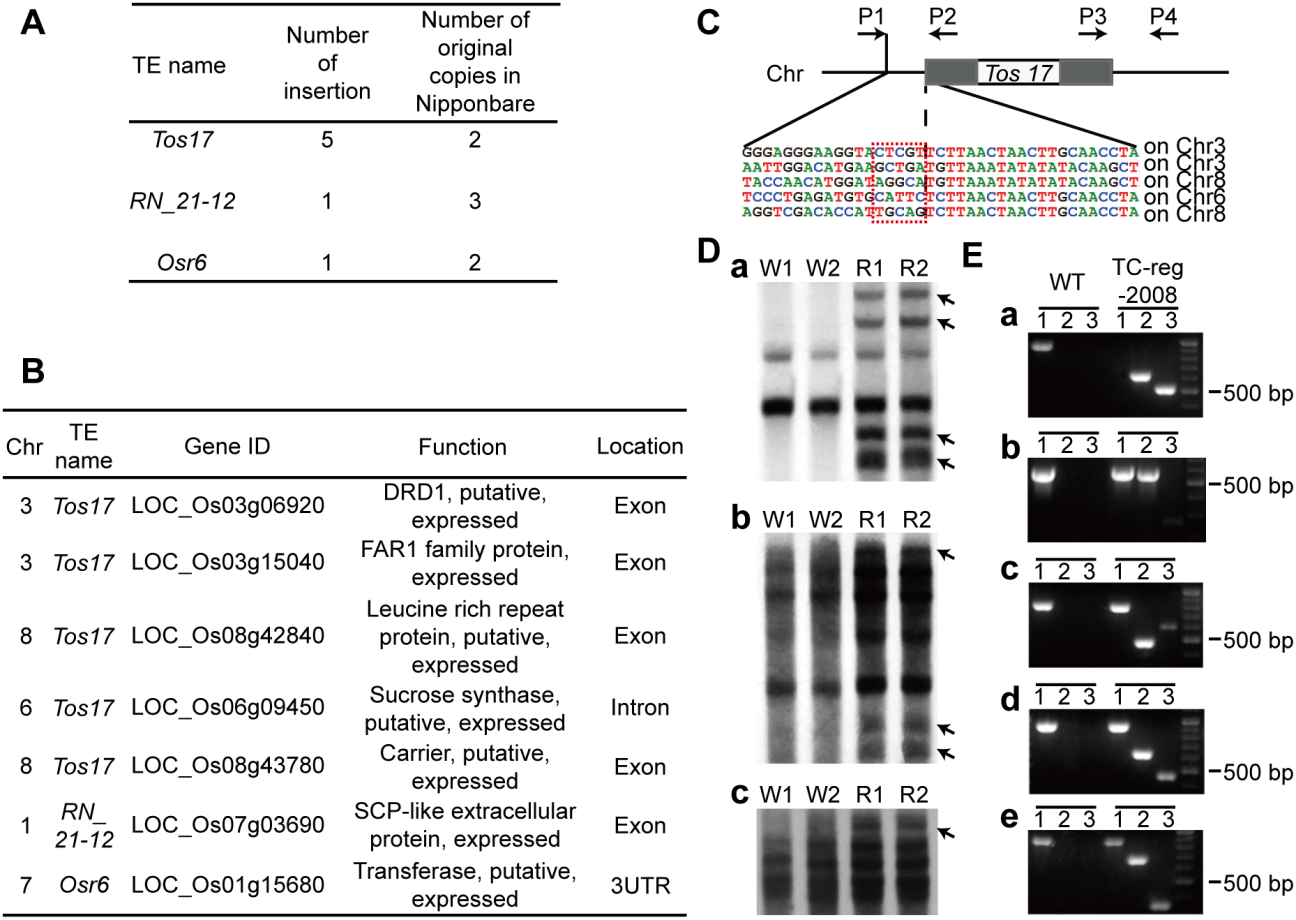
Detection and validation of TE mobility. (A) Three active retrotransposons and seven insertions detected by paired-end mapping based on genome re-sequencing. (B) Insertion positions of all the activated retrotransposons and the function analysis of affected genes in TC-reg-2008. (C) Location of primers designed for locus specific-PCR and the junctions of the *Tos17* insertion sites detected by Sanger-sequencing. P1/P4, P1/P2 and P3/P4 were three pairs of primers for amplifying the insertions of *Tos17*. The grey rectangles stand for LTRs of *Tos17*. The target site duplications of *Tos17* were boxed in red based on Sanger-sequencing. (D) Independent validation of the mobilization events by Southern blotting. a. *Tos17* probe, b. *RN_21–12* probe, c. *Osr6* probe. W1 and W2 stand for individual plants of wild type (WT), and R1 and R2 stand for individual plants of TC-reg-2008. (E) Five insertions by retrotransposon *Tos17* were validated by locus-specific PCR. Lanes 1, 2 and 3 represent the PCR amplicons by primers P1/P4, P1/P2 and P3/P4, respectively.

Transposable elements (TEs) are major constituents of plant genomes, which are usually epigenetically silenced by DNA methylation [22]. For example, >50% cytosines of rice TEs were methylated [23]. Under certain circumstances, some of the epigenetically silenced TEs can be reactivated to transpose and may lead to a variety of mutations by insertion disruption of gene function and/or re-modulating expression of TE-adjacent genes [24]. Tissue culture induced TE activity has been widely reported in various plant species [7], [25]–[38]. We therefore analyzed TE mobility in TC-reg-2008 by pairs of reads in which one read was mapped to a TE and the other was uniquely mapped on the rice genome (rationale detailed in Materials and methods). We detected seven new insertions caused by three retrotransposons inTC-reg-2008 (Figure 3A). Notably, all seven insertions occurred at genic regions, and of which five occurred in exons of five different genes, while one in intron of one gene and one in 3′UTR of another gene (Figure 3B). To independently validate these retrotranspositional events, we performed Southern blotting to test for copy number variation of the TEs, and/or by locus-specific PCR amplifications to test for new insertions by the most active retrotransposon, *Tos17* (Figure 3C). The retrotranspositional events by all three TEs were validated by Southern blotting, which showed increase of copy numbers consistent with retrotranspositions (Figure 3D). The five insertions by *Tos17* were also validated by locus-specific PCR amplifications (Figure 3E). Moreover, Sanger-sequencing of the specifically amplified TE-flank junctions were found to contain the canonical target site duplications (TSDs) for *Tos17* (Figure 3C). Furthermore, three forward insertions and two reverse insertions were identified by Sanger-sequencing (Figure 3C).

**Figure 4:**
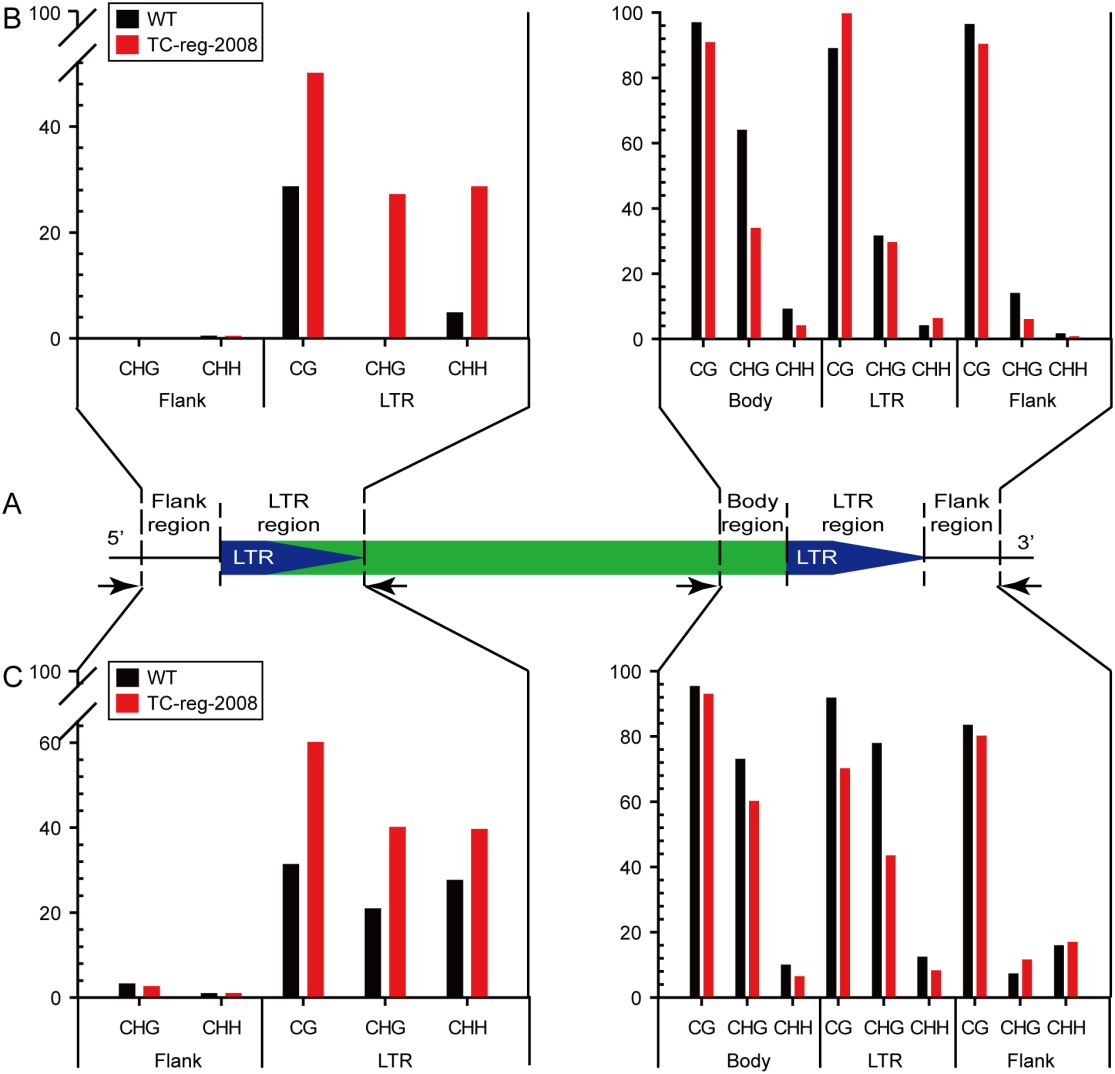
Tabulated changes in cytosine methylation of all three sequence contexts, CG, CHG and CHH at the 5′- and 3′-LTRs along with their contiguous flanks (for 5′- LTRs) or contiguous flanks and immediate body (for 3′-LTRs) in both original copies of *Tos17* in TC-reg-2008 *vs.* WT, based on bisulfite sequencing (for original data see Figure S4). (A) The schematic diagram of *Tos17* structure. The regions subjected to bisulfite sequencing were delineated. (B) Methylation changes of each sequence context in TC-reg-2008 (red bars) *vs.* WT (black bars) in the *Tos17* copy located on chromosome 10. (C) Methylation changes of each sequence context in TC-reg-2008 (red bars) *vs.* WT (black bars) in the *Tos17* copy located on chromosome 7.

TE activity is often causally linked to compromised repressive epigenetic states in which cytosine DNA methylation is a critical component. Prior studies have indicated extensive and transgenerational epigenetic remodeling associated with plant tissue culture [7], [13], [39]. To test if mobility of TEs detected in TC-reg-2008 occurred concomitantly with changes of cytosine methylation, we analyzed cytosine methylation patterns of the two original copies of *Tos17*, which showed the highest mobility in TC-reg-2008, by bisulfite sequencing (Figure 4; Figure S4). Specifically, we inspected the 5′ LTR along with a portion of its immediate upstream flank and the 3′LTR along with a portion of its upstream contiguous body region as well as downstream flanking region for each of the two *Tos17* original copies located on chromosomes 7 and 10, respectively, in TC-reg-2008 and WT (Figure 4A). We obtained the following results (Figure 4B, C; Figure S4A, B): (*i*) for both *Tos17* copies, the upstream flanks adjacent to the 5′LTRs were non-methylated in WT and TC-reg-2008; (*ii*) in WT, the 5′LTRs of both copies were moderately methylated at CG sites, but CHG and CHH sites showed clear differences between the two copies, with the copy located on chromosome 10 being non- or only slightly methylated while both sites of the copy located on chromosome 7 being methylated to similar extents as CG sites; (*iii*) in TC-reg-2008, both copies showed significant hypermethylation of all sequence contexts, CG, CHG and CHH; (*iv*) in both WT and TC-reg-2008, the 3′LTRs and their immediate upstream body-regions of both copies were heavily methylated at CG sites and moderately methylated at CHG and CHH sites, and in no case substantial difference was detected between the two genotypes for these regions of either copy; (*v*) in both WT and TC-reg-2008, the downstream flanks of both copies of *Tos17* showed heavy methylation at CG sites but little methylation at the CHG and CHH sites, and no difference existed between the two genotypes. Combined, it was evident that both original copies of *Tos17* had undergone cytosine methylation remodeling during and after the process of tissue culture; the hypermethylation detected primarily in their 5′LTRs (which are promoters for retrotransposon transcriptional expression) in the stabilized line TC-reg-2008 might reflect reinforced silencing via the RNA-directed DNA methylation (RdDM) pathway which has been shown as responsible for repressive control of *Tos17* [40]–[44].

## Discussion

In this study, genome-wide mutation rates, types and spectra induced by tissue culture in rice were analyzed in detail based on whole genome re-sequencing in a stabilized rice line (TC-reg-2008) derived from tissue culture followed by extensive selfing for eight successive generations. Our results are substantially different from excising results reported by previous studies in many aspects. We detected a mutation rate of 5.0×10^−5^ base substitutions per site, which is 21-fold and 29-fold higher than those reported in regenerants of *Arabidopsis* and a different genotypes of rice (Nipponbare), respectively [4], [6]. Moreover, a total of 16,936 small-scale Indels were detected in TC-reg-2008, which is dramatically higher than that in *A. thaliana* (only 21 were detected) [4], while Indels were not analyzed in the previous study in the other rice genotype Nipponbare [6]. Furthermore, seven mobilization events by three retrotransposons were identified and verified in this study, which were different from both previous studies in rice, as one of which showed only one TE (*Tos17*) being activated while the other showed 17 TEs being transposed [5]. No TE activity was found in regenerants of *Arabidopsis* [4]. Another notable difference between the tissue culture induced mutations we detected and those reported previously is random *vs*. biased chromosomal distribution of the mutations. We showed that both SNPs and small-scale Indels were non-randomly distributed across each of the12 rice chromosomes, and the same distribution trend was found between SNPs and Indels. In contrast, in the *Arabidopsis* study both SNPs (single base substitutions) and Indels were found to map evenly across the chromosomes [4]. In the previous rice study, SNPs were found to distribute randomly across the rice chromosomes while Indels were not analyzed [6]. The pattern of base substitution can be transitions (Ts) or transversions (Tv), and both types of substitution were detected in TC-reg-2008. The rate of transition to transversion we detected was 2.37, which was also different from those reported in the *Arabidopsis* and rice (cv. Nipponbare) regenerants [4], [6]. We note however that the within transition and transversion biases, i.e., G:C > A:T, were similar to many previous reports on naturally occurred mutations [45]–[47], possibly due to the highly conserved mechanism of deamination of methylated cytosines in all higher eukaryotes studied [47].

Taken together, it is clear that the types, rates, and spectra of tissue cultured induced mutagenesis are highly variable, which may vary across species, genotypes and conditions of tissue culture, or simply being fortuitous. Although we have shown previously based on DNA marker analysis that relative to epigenetic alteration, genetic mutation is the major type of molecular changes associated with rice tissue culture [48], both previous studies [7], [13], [39] and our data here showed that at least TE mobilization occurs concomitantly with DNA methylation dynamics, consistent with their repressive control by this epigenetic marker [22], [23], [40]. The involvement of epigenetic mechanisms lends support to the fortuitous aspect of tissue culture induced mutagenesis, as the later is known to occur stochastically [49].

Domestication-related regions of rice have been identified in previous studies, and which are genetically labile and prone to mutations under natural circumstances [15]. Here we show that the hyper-mutagenic genomic regions as a result of tissue culture showed little coincidence with these hyper-mutable regions under nature conditions. This suggests that the mutagenic mechanisms underlying natural mutation and tissue culture-induced mutagenesis might be different. Of course, we cannot rule out the possibility that it was long-term selection that makes the difference, as the somaclonal line (TC-reg-2008) we used has undergone only eight generations without purposeful selection. Still, we consider that the issue merits further investigation to unveil possible novel aspect of practical implications of tissue culture in crop improvements.

Most organisms have the ability to stabilize their phenotypes although they harbor abundant cryptic genetic variations via the mechanism of canalization [16], [17]. Nevertheless, the hidden genetic variations can be penetrated under certain stressful environments [21]. We have shown here that the large amount of homozygous genetic variations including those apparently altered functionality of important enzymes or proteins manifested no phenotypic effects in the tissue culture-derived line (TC-reg-2008) compared to WT under normal growing conditions. If without further investigation, gene redundancy might be considered as a plausible explanation for this phenomenon. However, we demonstrated that significant phenotypic variations were observed between TC-reg-2008 and WT when the plants were grown under several abiotic stress conditions. This suggests that gene functional redundancy cannot be the only cause for the cryptic nature of the genetic variations. Instead, perturbation of canalization by the stresses, and hence penetrance of the genetic variations should be at least partially responsible for the phenomenon.

## Supporting Information

Figure S1 **Correlation of SNPs and Indels per 100 kb interval on the whole genome level and for each of the 12 rice chromosomes.** The *X*- and *Y*-axes stand for the number of SNPs per 100 kb window and the number of Indels per 100 kb window in TC-reg-2008, respectively.
(TIF)

Figure S2 **Distribution of DNA polymorphisms within 100 kb window across each chromosome.** The *X*-axis represents the physical distance along each chromosome, splitting into 100-kb sliding windows. The *Y*-axis indicates the number of SNPs, insertions or deletions. The black dot in each chromosome stands for centromere. The two boxed regions represent the domestication related regions identified inTC-reg-2008.
(TIF)

Figure S3 **Phenotypic variations induced by different abiotic stresses between TC-reg2008 and its WT.** (A) Photographed phenotypes in different stress conditions. (a)–(e) are control, salt, CuSO_4_, HgCl_2_ and overdose NO, respectively. Scale bars are 3 cm. (B)–(D) Tabulated results of shoot length, root length and fresh weight between TC-reg-2008 and its WT under different stresses. The black and grey vertical bars denote WT and TC-reg-2008, respectively. **indicate statistical significance at the 0.01 statistical level (One-way ANOVA).
(TIF)

Figure S4 **Bisulfite sequencing-based cytosine methylation maps of the two Tos17 original copies located on chromosome 10 and chromosome 7, respectively.** (A) All three types of cytosines, CG (red circles), CHG (blue circles) and CHH (green circles) generated by bisulfite sequencing for (a) the upstream flank and 5′LTR of *Tos17* and (b) the 3′LTR along with a portion of its upstream contiguous body region and downstream flank of *Tos17* on chromosome 10 for both WT and TC-reg-2008. (B) The same as above for (a) the upstream flank and 5′LTR of *Tos17* and (b) the 3′LTR along with a portion of its upstream contiguous body region and downstream flank of *Tos17* on chromosome 7 for both WT and TC-reg-2008.
(TIF)

Table S1 **A list of primers used for amplifying the Southern blotting probes.**
(DOC)

Table S2 **A list of primers used for locus-specific PCR.**
(DOC)

Table S3 **A list of primers used for bisulfite sequencing.**
(DOC)

Table S4 **Number of Illumina GA reads and coverage of the Nipponbare genome.**
(DOC)

Table S5 **Classification of base substitution mutations detected for each chromosome.**
(XLS)

Table S6 **List of all nonsynonymous SNPs.**
(XLS)

Table S7 **List of large effect SNPs.**
(XLS)
