# Supplementary figures and images for: Tissue Culture-Induced Heritable Genomic Variation in Rice, and Their Phenotypic Implications
Source: PLoS One. 2014 May 7;9(5):e96879. doi: 10.1371/journal.pone.0096879 (PMC4013045; doi:10.1371/journal.pone.0096879)

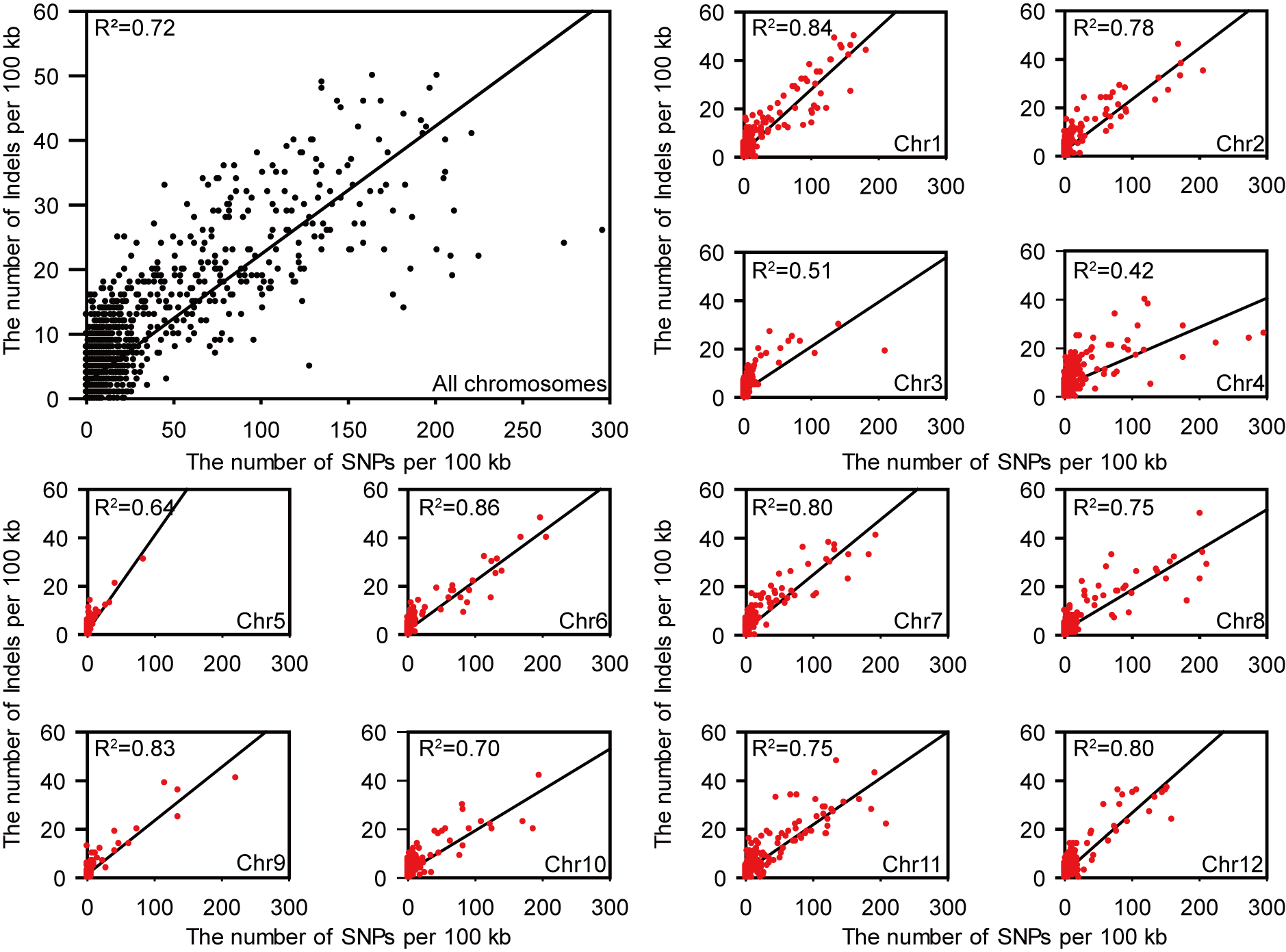

Supplement: Figure S1 — Correlation of SNPs and Indels per 100 kb interval on the whole genome level and for each of the 12 rice chromosomes. The X- and Y-axes stand for the number of SNPs per 100 kb window and the number of Indels per 100 kb window in TC-reg-2008, respectively. (TIF) [file pone.0096879.s001.tif]

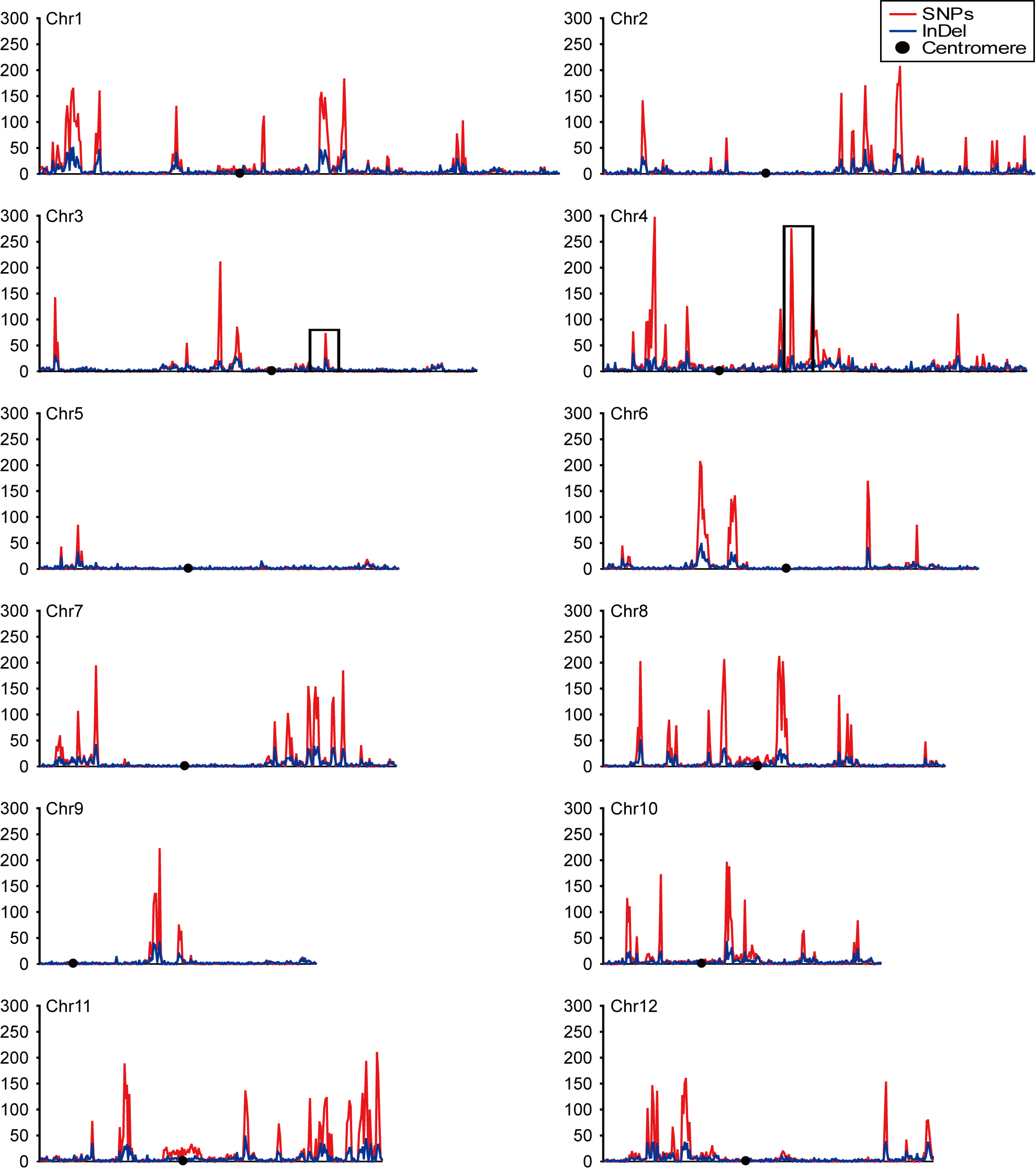

Supplement: Figure S2 — Distribution of DNA polymorphisms within 100 kb window across each chromosome. The X-axis represents the physical distance along each chromosome, splitting into 100-kb sliding windows. The Y-axis indicates the number of SNPs, insertions or deletions. The black dot in each chromosome stands for centromere. The two boxed regions represent the domestication related regions identified inTC-reg-2008. (TIF) [file pone.0096879.s002.tif]

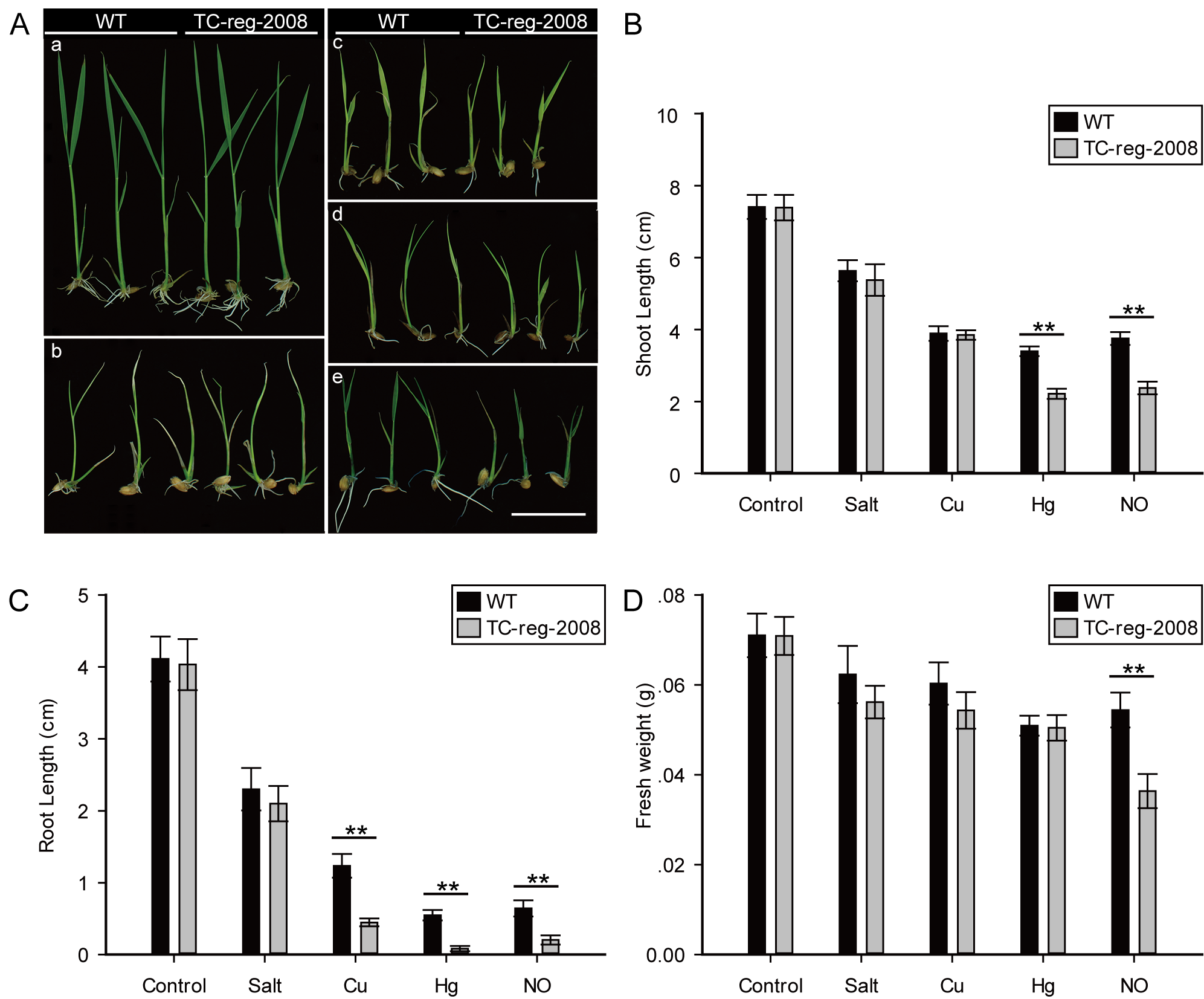

Supplement: Figure S3 — Phenotypic variations induced by different abiotic stresses between TC-reg2008 and its WT. (A) Photographed phenotypes in different stress conditions. (a)–(e) are control, salt, CuSO4, HgCl2 and overdose NO, respectively. Scale bars are 3 cm. (B)–(D) Tabulated results of shoot length, root length and fresh weight between TC-reg-2008 and its WT under different stresses. The black and grey vertical bars denote WT and TC-reg-2008, respectively. **indicate statistical significance at the 0.01 statistical level (One-way ANOVA). (TIF) [file pone.0096879.s003.tif]

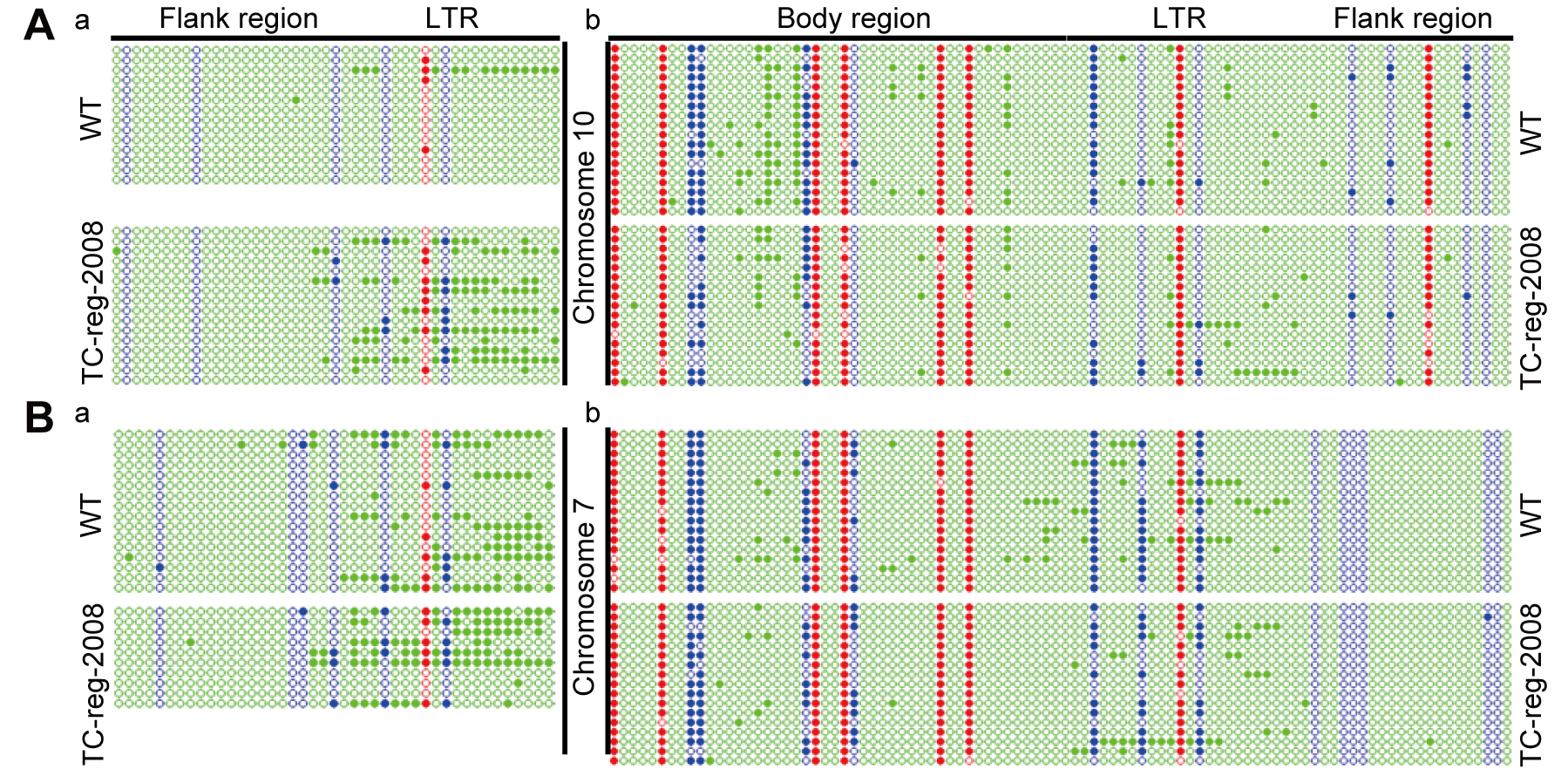

Supplement: Figure S4 — Bisulfite sequencing-based cytosine methylation maps of the two Tos17 original copies located on chromosome 10 and chromosome 7, respectively. (A) All three types of cytosines, CG (red circles), CHG (blue circles) and CHH (green circles) generated by bisulfite sequencing for (a) the upstream flank and 5′LTR of Tos17 and (b) the 3′LTR along with a portion of its upstream contiguous body region and downstream flank of Tos17 on chromosome 10 for both WT and TC-reg-2008. (B) The same as above for (a) the upstream flank and 5′LTR of Tos17 and (b) the 3′LTR along with a portion of its upstream contiguous body region and downstream flank of Tos17 on chromosome 7 for both WT and TC-reg-2008. (TIF) [file pone.0096879.s004.tif]
