# Supplementary material for: Tissue Culture-Induced Heritable Genomic Variation in Rice, and Their Phenotypic Implications
Source: PLoS One. 2014 May 7;9(5):e96879. doi: 10.1371/journal.pone.0096879 (PMC4013045; doi:10.1371/journal.pone.0096879)
Supplement: Table S1 — A list of primers used for amplifying the Southern blotting probes. (DOC) [file pone.0096879.s005.doc]

**Table S1. A list of primers used for amplifying the Southern blotting probes.**

| **Primer Name** | **Sequence** |
| --- | --- |
| *Tos 17*_sense | GCTACCCGTTCTTGGACTAT |
| *Tos 17*_antisense | CTGAAATCGGAGCACTGACA |
| *RN_21-12*-sense | GCTGAACGGGAGGATGAAT |
| *RN_21-12*-antisese | CGTGAGTTGTGGGTAGGAGAA |
| *Osr6*_sense | CGCATTACTTCCGCTCGTGT |
| *Osr6*_antisense | CGCCTCTTTGCCCTTTGG |
