# Supplementary material for: Tissue Culture-Induced Heritable Genomic Variation in Rice, and Their Phenotypic Implications
Source: PLoS One. 2014 May 7;9(5):e96879. doi: 10.1371/journal.pone.0096879 (PMC4013045; doi:10.1371/journal.pone.0096879)
Supplement: Table S2 — A list of primers used for locus-specific PCR. (DOC) [file pone.0096879.s006.doc]

**Table S2.** A list of primers used for locus-specific PCR.

| Primer Name | Sequence |
| --- | --- |
| Tos17_LTR_5 | ATGTACTGTATAGTTGGCCCATGTC |
| Tos17_LTR_3 | CATGGGCCAACTATACAGTACATTAG |
| Tos17_Chr3-1_Flanking_sense | GATGGACAAATCTCGTGCTA |
| Tos17_Chr3-1_Flanking_antisense | CCTCGCTATCTGAAACATCCA |
| Tos17_Chr3-2_Flanking_sense | GCATTATCCAAATAGTTGTTCCAG |
| Tos17_Chr3-2_Flanking_antisense | TTGGCTCATTGTCTTGATTTG |
| Tos17_Chr8-1_Flanking_sense | TCCTGATATGCCAAATAGTGT |
| Tos17_Chr8-1_Flanking_antisense | TCCTATTTCACTGGGTATCTCC |
| Tos17_Chr6_Flanking_sense | TATCAGCAGCCTTGTCACT |
| Tos17_Chr6_Flanking_antisense | CGTCCCTGTTATTCACCTAC |
| Tos17_Chr8-2_Flanking_sense | ATGTCAATGGACCAAGGCTG |
| Tos17_Chr8-2_Flanking_antisense | TTGCTCCACTGCTTTCCTG |
