# Supplementary material for: Tissue Culture-Induced Heritable Genomic Variation in Rice, and Their Phenotypic Implications
Source: PLoS One. 2014 May 7;9(5):e96879. doi: 10.1371/journal.pone.0096879 (PMC4013045; doi:10.1371/journal.pone.0096879)
Supplement: Table S3 — A list of primers used for bisulfite sequencing. (DOC) [file pone.0096879.s007.doc]

**Table S3. A list of primers used for bisulfite sequencing.**

| **Primer Name** | **Sequence** |
| --- | --- |
| Tos17_Chr10-5-F1 | GTTGGGATYAATGGGATTGGYAAGT |
| Tos17_Chr10-5-R1 | CCTCRACCTRTRCARCAARCCARCAAC |
| Tos17_Chr10-5-F2 | TGGAYAGATYAAGYYTAAYTTGGGAAG |
| Tos17_Chr10-5-R2 | RACCATTRCTCTRATACCATCTTAACT |
| Tos17_Chr10-3-F1 | AYAAYYAYTTYAGAGATTGTGTGGTTG |
| Tos17_Chr10-3-R1 | CARCARTTTATTTACATRATRATACA |
| Tos17_Chr10-3-F2 | YGGAGYTATAYAAATYGYYAATGAT |
| Tos17_Chr10-3-R2 | RCAATCRARTAAAAAAACATRCACCT |
| Tos17_Chr7-5-F | YTYGGATGTYTTTAGATGTAYTTAAAAAAGG |
| Tos17_Chr7-5-R | TARCCCACRARRCRACRRTRAAAARRACA |
| Tos17_Chr7-3-F | ATGATYYAATYAAGYATGAATTGAYGAAGYATATTG |
| Tos17_Chr7-3-R | AAARTTCARATRCTTRTTCACRTCTTCATRCAT |
