# Supplementary material for: Tissue Culture-Induced Heritable Genomic Variation in Rice, and Their Phenotypic Implications
Source: PLoS One. 2014 May 7;9(5):e96879. doi: 10.1371/journal.pone.0096879 (PMC4013045; doi:10.1371/journal.pone.0096879)
Supplement: Table S4 — Number of Illumina GA reads and coverage of the Nipponbare genome. (DOC) [file pone.0096879.s008.doc]

**Table S4.** Number of Illumina GA reads and coverage of the Nipponbare genome.

|  | **cv. Hitombare** | **Regenerated rice** |
| --- | --- | --- |
| FASTQ | 78430688 | 80792510 |
| Unique map | 59181778 | 61841287 |
| Multiple map | 14521790 | 14682205 |
| Unmapped | 4727120 | 4269018 |
| Effective fold genome^a^ | 14.7 | 15.4 |
| ^a^ Fold genome was calculated based on the Map data, the reference genome of Nipponbare (MSU 7.0) was 373245519 bp | | |
